# The Isolation and Assessment of the Levels of Microbial Pathogens From Saliva, Hands, and Currency Notes

**DOI:** 10.7759/cureus.74366

**Published:** 2024-11-24

**Authors:** Mythili Sabesan, Divyashree Venkatesan

**Affiliations:** 1 Oral Pathology and Microbiology, Sri Ramachandra Dental College and Hospital, Sri Ramachandra Institute of Higher Education and Research, Chennai, IND; 2 Medicine, Sri Ramachandra Dental College and Hospital, Sri Ramachandra Institute of Higher Education and Research, Chennai, IND

**Keywords:** cross-contamination, culture, currency notes, microbial pathogens, saliva

## Abstract

Introduction

Currencies such as coins and paper notes are always used for the purchase of products and services by the common man. These currencies are constantly being subjected to contamination and thus serve as a potential medium for the transfer of microbial pathogens. Thus, this study aims to establish public awareness of the proper handling of currency notes to prevent cross-contamination and the incidence of infectious diseases.

Methods

This study aims to identify and evaluate the levels of microbial pathogens in the saliva, hands, and currency notes from inoculated culture plates.

Result

On evaluation of the cultured plates, the presence of microbial pathogens was evident at 24 hours and 48 hours of growth. Staining of the sub-cultured plates aided in the identification of the organisms.

Conclusion

This study concludes that contaminated currency notes can act as a vehicle enabling the transmission of microbial pathogens and the incidence of infectious diseases.

## Introduction

Currencies such as coins and paper are most commonly used as a medium as now we have more digital forms for the purchase of products and services by the common man. Though digital banking has become more popular these days, currencies are still being used widely during deposit and withdrawal in banks and post offices. These notes, when continually used, serve as a contaminated vehicle for the transmission of various pathogenic organisms [1]. Simultaneous handling of paper currency notes along with food in public places could cause transmission of microorganisms and can lead to contamination of food [2]. The contamination of currency notes takes place at various stages during their use, which includes production, handling, usage, and finally, storage of these notes under various conditions [3]. Various factors determining the levels of contamination include lower denomination currencies, the material of the currency notes, and the age of the currency notes [4]. Thus, when these contaminated currency notes are being used, the rate of incidence of infectious diseases increases [5].

Microbial contamination can occur through direct transmission or indirect transfer via contact through hand-to-hand contact, food, and other inanimate objects. These routes of transmission play a major role in the health of many populations in developing countries [6]. The contamination of various pathogenic microorganisms is known to be responsible for the manifestations of watery diarrhea, oral and skin diseases, gastrointestinal disturbances, and respiratory diseases like pneumonia [7]. A study by Matel et al. aimed at assessing the pathogenic organisms from currency notes obtained from various populations, namely fruit sellers, butchers, hospitals, and banks, concluded that contamination of currency notes aids in the transmission of infectious diseases [8].

Researchers at the Regional Sophisticated Instrumentation Centre (RSIC) at the North Eastern University in Shillong, India, assessed Indian banknotes and identified microbial organisms causing diseases like tuberculosis, meningitis, and infections of the tonsils, genital tract, and gastrointestinal tract. The organisms from the currency notes infect the human body through scratches on the hands or when humans touch their nose and mouth with contaminated hands [9]. This contamination could be prevented by periodic disinfection of currency notes at the source by ultraviolet light and fumigation. Also, it is recommended to remove worn-out or mutilated currency by concerned authorities as prolonged usage can lead to more contamination [2]. Public awareness and emphasis on currency notes being the source of infection, the role of personal hygiene, regular microbial testing, and periodic disinfection of the currency notes would go a long way in preventing the spread of pathogenic microbes [10].

Thus, the aim of the current study was to isolate and assess the levels of microbial pathogens in saliva, hands, and currency notes from the cultured plates.

## Materials and methods

Objectives

The primary objective of this study was the isolation of *Staphylococcus aureus*, *Salmonella typhi*, *Escherichia coli*, and *Pseudomonas aeruginosa* from saliva, hands, and currency notes. The levels of *Staphylococcus aureus*, *Salmonella typhi*, *Escherichia coli*, and Pseudomonas aeruginosa were then assessed from the cultured plates.

Materials

The materials used for collecting and transporting saliva samples and smears include UV-sterilized currency notes, sterile cotton swabs, absolute alcohol, nutrient broth, sterile packets, and saliva containers and labels. For culturing the samples, the medium required included MacConkey agar, blood agar, and brain heart infusion (BHI) broth for sub-culture. 

Methodology

Ethical Clearance

The current study is an observational study that was presented and approved by the Institutional Ethics Committee (CSP/21/MAY/94/361). The study was conducted for a period of four months, from August 2021 to November 2021. The study samples were collected from healthy attendees of patients of the Sri Ramachandra Faculty of Dental Sciences. The samples were then labeled and transported to the laboratory following standard protocols. Based on previous studies, the number of participants for the study was calculated as 20.

Sample size calculation

Sample size was calculated using the following formula: n = (Z^2^x P x q)/ d^2^, where Z = 1.96, P = prevalence rate of 0.64, q = (1-p) = 1 - 0.64; and d = 20% of p.

Sample size

The sample size included the following: 10 of 10 rupees currency notes, 20 participants, 10 control samples, 40 samples from currency notes after handling (two broths with one sample each), 40 samples of hands, and 20 salivary samples. The total sample size was 110.

Inclusion and Exclusion Criteria

The inclusion criteria included healthy participants in the age group of 18-45 years of age who are attenders of patients reporting to Sri Ramachandra Faculty of Dental Sciences, currency notes printed after demonetization, and participants' willingness. The exclusion criteria included participants under any kind of medication.

Procedure

Preparation of currency notes: The collected currency notes were UV sterilized at the site of sample collection by the Trendz UV machine, dropped into a sterile polythene bag, and labeled accordingly. Currency notes were not handled by the investigator using bare hands at any stage.

Sample collection: Control samples were collected from the sterilized currency notes before handling. Then, the participants were advised to count the currency notes multiple times. After handling, samples were collected from the hands of the participants using sterile cotton swabs dipped in nutrient broth. Samples were collected using sterile cotton swabs dipped in nutrient broth from the contaminated currency notes. For saliva samples, participants were advised to gargle with water, collect saliva on the floor of the mouth for one minute, and spit into sterile saliva containers. The samples were then labeled and transported in ice containers to the laboratory for further analysis.

Sample analysis: The samples collected were inoculated onto MacConkey agar and blood agar, respectively. Sterile BHI broth was added to the collected sample. All the plates and BHI broths were incubated at 37°c for 24 hours. The plates that were inoculated on the previous day were observed for growth. After doing gram staining from the growth, they were processed in the Vitek 2 system (bioMérieux, Marcy-l'Étoile, France) for identification of the organism. All the BHI broths were subcultured (inoculated) onto MacConkey and Blood agar plates and incubated at 37°c for 24 hours. All the sub-culture plates were observed for growth on the next day. After doing gram staining from the growth, they were processed in the Vitek 2 system for identification of the organism.

## Results

**Figure 1:**
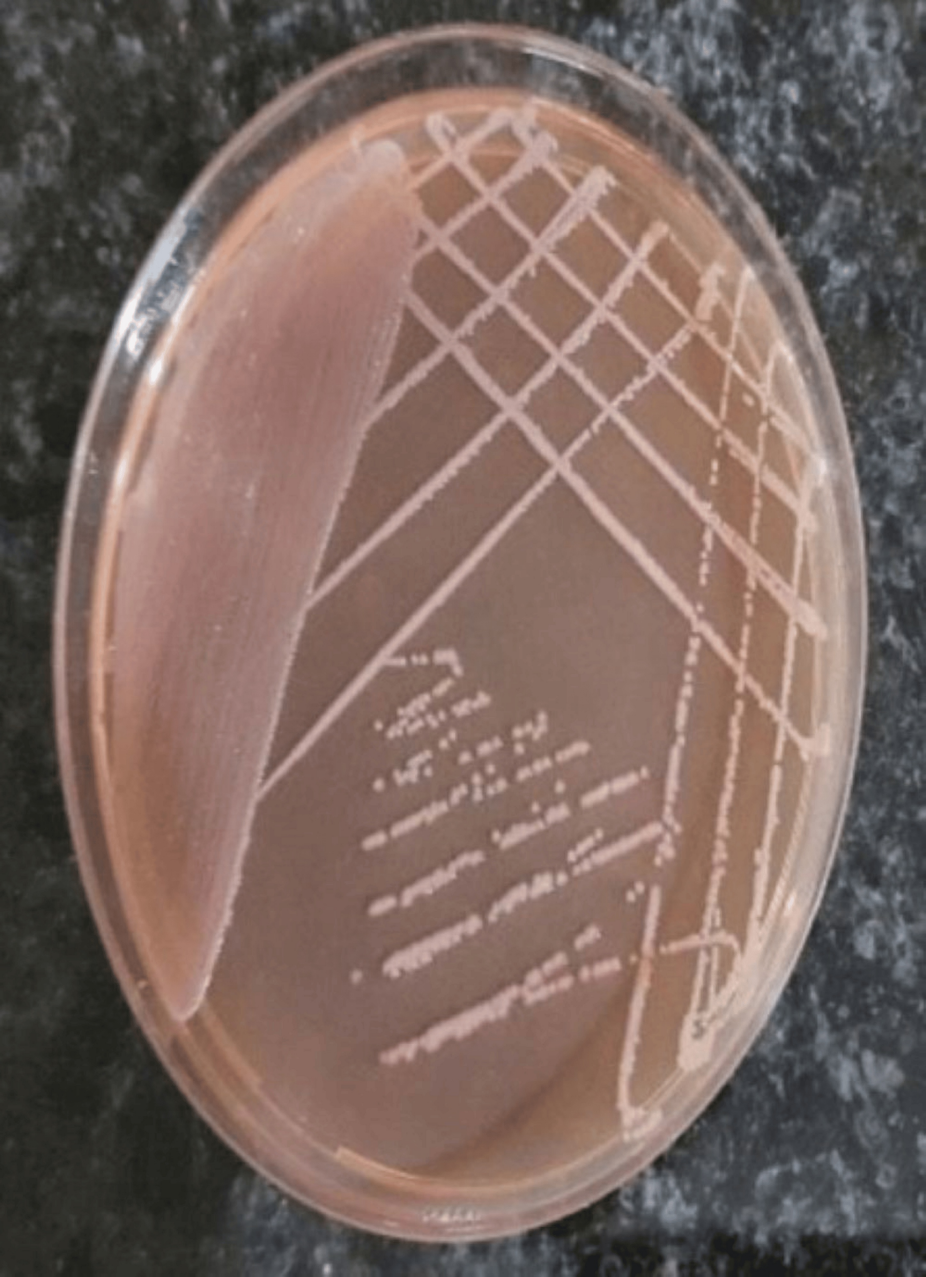
Acinetobacter baumannii

**Figure 2:**
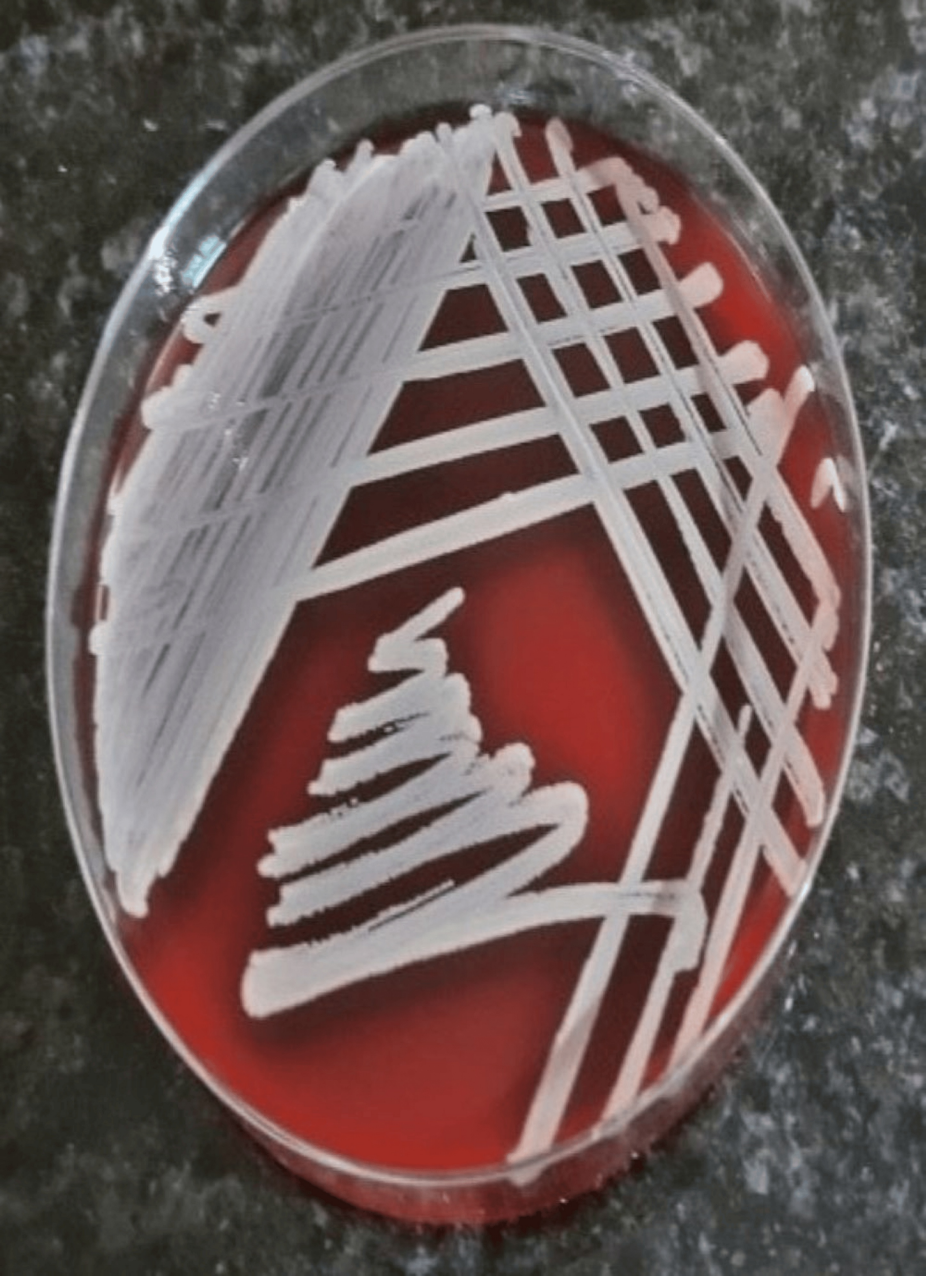
Staphylococcus aureus

**Figure 3:**
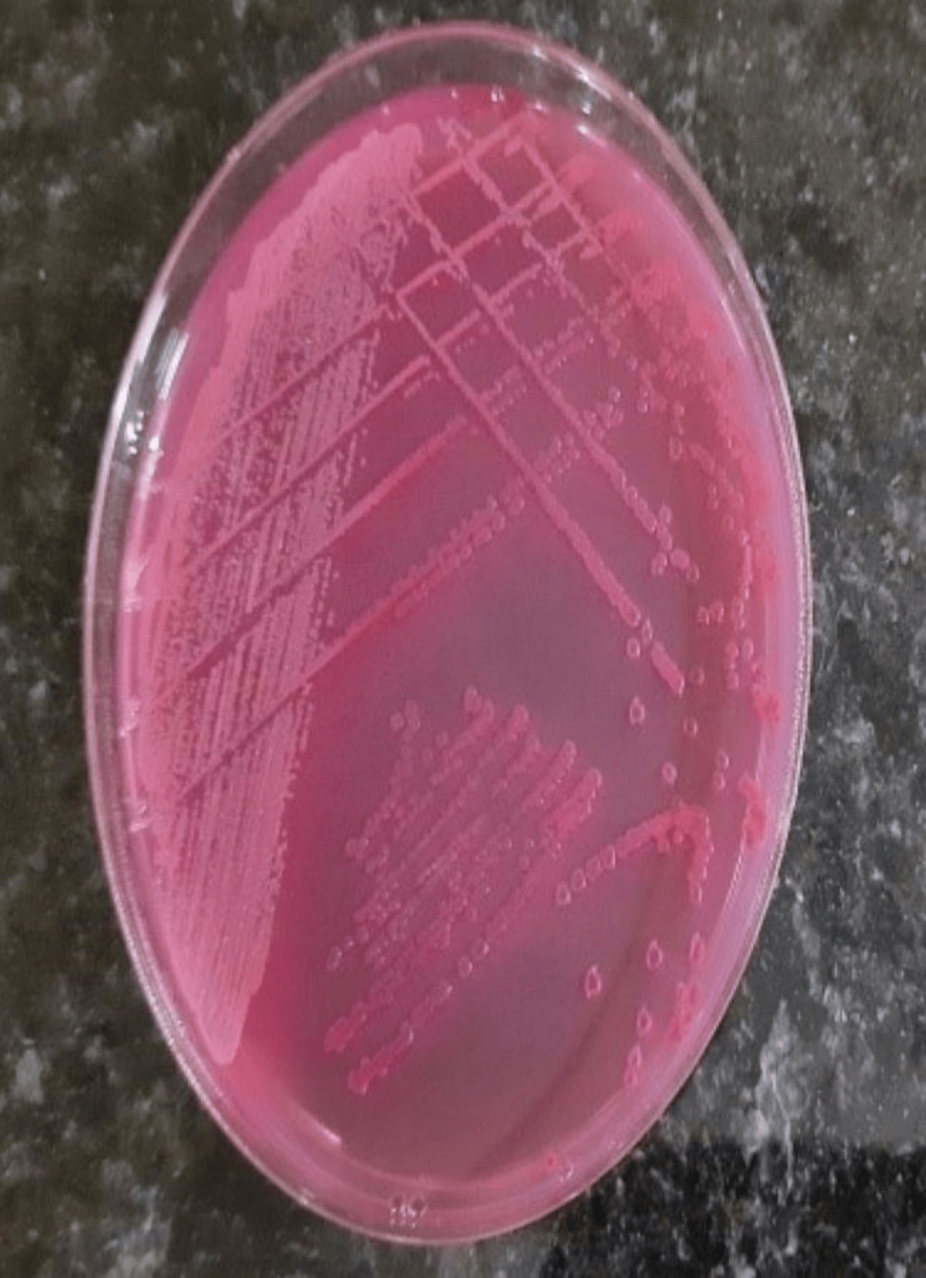
Escherichia coli

**Figure 4:**
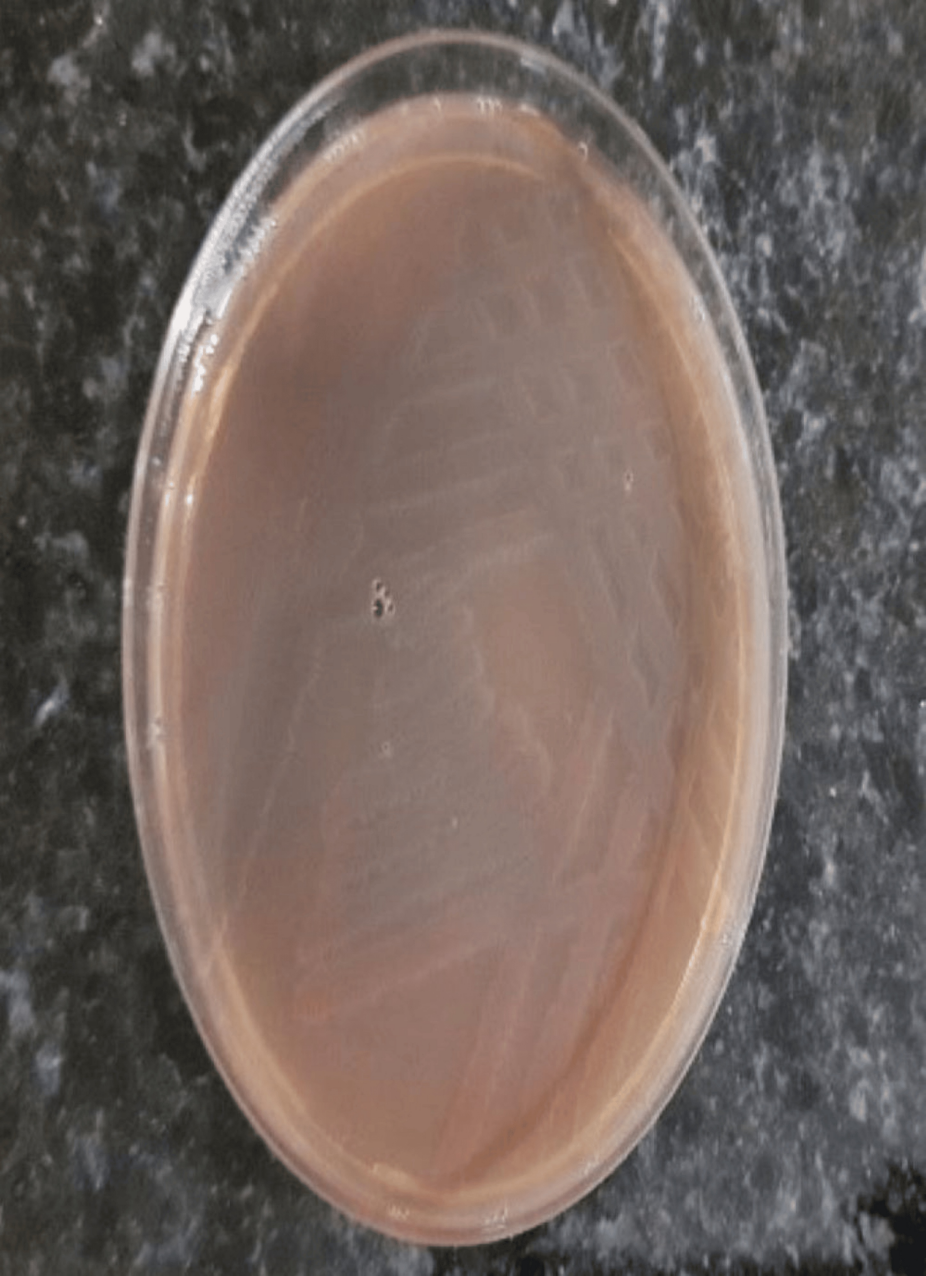
Pseudomonas aeruginosa

The current study involved the participation of 20 healthy volunteers who were asked to handle 10 sterile currency notes. On examining the cultured samples taken from each of the participant's hands, saliva, and currency notes, the presence of microbial pathogens was evident at 24 hours and 48 hours of growth. Table 1 shows the growth of organisms at 24 hours and 48 hours, respectively. The assessment of levels of organisms revealed decreased growth at 24 hours when compared with 48 hours of growth. Upon sub-culturing, that is after 48 hours, various microbial pathogens were found to be present, such as gram-positive, gram-negative, and aerobes. A total of 75% of the observed samples from saliva were positive for the presence of microbes, of which only one was non-pathogenic (*Staphylococcus epidermis*), 60% of the samples from hand were positive for the presence of microbes with one non-pathogenic organism (*Staphylococcus epidermis*), 35% of the samples from currency notes were positive for microbes, and all were pathogenic (Table 1). All the 10 control samples that were cultured showed no growth even after 48 hours. Identification showed the active participation of *Staphylococcus aureus*, *Escherichia coli*, and *Acinetobacter baumannii *on the whole (Table 2). Figure 1 shows the growth of *Acinetobacter *at 48 hours from the samples. Figures 2-4 show the growth of organisms *Staphylococcus aureus*, *Escherichia coli*, and *Pseudomonas aeruginosa *from the samples.

**Table 1:** Assessment of levels of microbes in the cultures and sub-culture at 24 hours and 48 hours

Organism	After 24 hours			After 48 hours		
	Saliva	Currency	Hands	Saliva	Currency	Hands
Staphylococcus aureus	1	0	1	2	3	3
Enterococci	0	1	0	3	0	0
Staphylococcus epidermidis	0	0	0	1	0	1
Escherichia coli	2	0	3	1	1	1
Klebsiella pneumoniae	1	0	0	8	0	2
Pseudomonas aeruginosa	0	0	0	2	2	0
Enterobacter cloacae	0	0	0	1	0	2
Acinetobacter baumanni	0	0	0	3	2	3

**Table 2:** Total number of positive samples for specific organisms

Organisms	Total		
	Saliva	Currency	Hands
Staphylococcus aureus	3	3	4
Enterococci	3	1	0
Staphylococcus epidermidis	1	0	1
Enterobacter cloacae	1	0	2
Acinetobacter baumanni	3	2	3

## Discussion

The current study is one of the few studies on the contamination of currency notes carried out in our Indian population. The isolation of microbial pathogens from saliva, hand, and currency notes, as demonstrated by the current study, shows that currency notes could play an important role in the transmission of microbial pathogens in the community and thus present a public health threat. The growth of Staphylococcus aureus, Acinetobacter, and Escherichia coli organisms in the cultures and sub-cultures was substantially increased. The presence of cross-contamination is seen by *Staphylococcus aureus*, *Acinetobacter*, and *Escherichia coli*. The presence of *Escherichia coli *is an indication of fecal contamination and poor personal hygiene practices of currency handlers. This organism is known to cause food poisoning, urinary tract infections, and in severe cases, pneumonia. However, *Staphylococcus aureus *causes abscesses, cellulitis, bloodstream infections, etc. According to a study done by Kumar et al., *Staphylococcus aureus* can survive on paper notes for eight days. Prolonged survival of this pathogen on currency permits transmission [11]. Acinetobacter baumannii causes blood, urinary tract, and lung infections. Also, it can colonize in the respiratory secretions. Thus, simultaneous handling of money and food should be discouraged. The number of samples indicative of cross-contamination was smaller in our study as the study population taken was small. However, the presence of microbial pathogens, as seen in our study, suggests currency could play a role in cross-contamination of diseases. Another aspect of bacterial contamination is directly related to the adherence of organisms in the bank notes. This adherence is associated with the material of the currency notes [5].

Limitations 

The current did not include the analysis of cross-contamination of currencies owing to the smaller sample size of the study. Thus, if the study is carried out on a larger sample size, a proper correlation of cross-contamination can be made. Also, the current study does not extend to associate the adherence of bacteria and the material of the currency notes.

## Conclusions

The current study concludes that currency notes can act as a mode of transmission of infectious diseases and can lead to cross-contamination, especially in places like hospital set-ups, from the result presented above. The unhygienic practice of most people includes the wetting of hands or fingers with saliva or the use of contaminated water to lubricate the hand in counting money and the use of food-contaminated fingers in handling currency notes, which can increase the risk of cross-contamination. Regular disinfection of currency deposited in banks by ultraviolet light or formalin vapors can prevent the transmission of these pathogens. Emphasizing and public awareness of proper handling of currency notes will go a long way in preventing cross contamination and incidence of infectious diseases.
